# Supplementary material for: Keratins as an Inflammation Trigger Point in Epidermolysis Bullosa Simplex
Source: Int J Mol Sci. 2021 Nov 18;22(22):12446. doi: 10.3390/ijms222212446 (PMC8624175; doi:10.3390/ijms222212446)
Supplement: Supplementary file 1 [file ijms-22-12446-s001.zip › ijms-1397834-supplementary.pdf]

Supplementary Table S1

EBS subtype, non-syndromic	ORPHA\OMIM	Gene	Inheritance	Clinical features	References
EBS, localized **EBS loc.**	79400\131800	*KRT5* (more prevalent) or *KRT14*	AD	Blisters localized mainly on the palms and the soles, life-expectancy is normal	[111]
**EBS** - AD-intermediate,	79399\131900	*KRT5* or *KRT14*	AD	Blistering generalized, not severe, conditions worsen in warm weather, absence of tonofilaments in basal KCs on electron microscopy	[8]
EBS severe, **EBS-sev**	79396\131760	*KRT5* or *KRT14*	AD	Early onset of generalized and widespread blistering, tense herpetiform blisters, nail dystrophy, palmoplantar keratoderma; hyper and hypopigmentation	[38,44]

				with generalized and small blisters in grouped or arcuate configuration	
EBS with mottled pigmentation **EBS-MP**	79397\131960	*KRT5* (often) or *KRT14* (less frequently)	AD	Upper trunk, neck and acral blistering early in childhood, mottled or reticular pigmentation distributed in a number of sites, focal punctate hyperkeratosis of the palms and soles	[208]
EBS, severe **EBS AR sev**	89838\601001	*KRT14*	AR	Rare variant. Generalized blistering, intermediate or severe, atrophic scarring and dystrophic nails usually occur, along with focal keratoderma (palms and soles). Severe generalized blistering may cause perinatal death or persist during the entire life.	[209,210]

EBS intermediate with exophilin 5 deficiency **EBS-AR-EXPH5**	412189\615028	*EXPH5*	AR	Generalized or localized intermittent blistering, skin fragility, slight scarring and post-inflammatory hyperpigmentation. Clinical symptoms improve with age.	[57,211]
EBS intermediate with PLEC mutations **EBS-AD-PLEC** **(EBS-Ogna)**	79401\131950	*PLEC*	AD	Primarily acral blistering, bruisability, hemorrhagic blistering, and onychogryphosis.	[67]
**EBS AR** KRT14	89838\601001	*KRT14*	AR	Generalized or, less frequently, localized acral blistering with early onset, palmo-plantar hyperkeratosis.	[209]
		KRT5			[52]
PLEC		*PLEC*		Early mortality<br>Intermediate, congenital myopathy developed after mild skin blistering in infancy.	[212,213]

EBS with migratory circinate, **EBS-migr**	158681\609352	*KRT5*	AD	Belt-like areas of erythema with multiple vesicles and small blisters, heal with brown pigmentation but no scarring	[214,215]
EBS due to plakophilin deficiency, **EBS-PD**	158668\604536	*PKP1*	AR	Suprabasal subtype of EBS, characterized by generalized superficial erosions and less commonly blistering.	[216]
EBS localized due to BP230 deficiency, EBS-DST related	412181\615425	*DST*	AR	Relatively mild blistering with acral predominance, plantar keratoderma.	[217]
EBS confined to the hands and feet	147557\131800	*ITGB4*	AD	Mild blistering of hands and feet from birth, dystrophy of the nails with onychogryphosis, and enamel hypoplasia. The level of blistering was intraepidermal.	[218]

**Group of disorder Syndromic EBS types ORPHA 595351**<br><br>Epidermolysis bullosa simplex with extracutaneous involvement					
EBS intermediate with muscular dystrophy, EBS-MD	257\226670	*PLEC*	AR	Intermediate form with generalized blistering with mucosal involvement. In addition to skin symptoms, *PLEC* mutations cause abnormal interaction with desmin. Muscle disease with variable onset.	[66]
EBS severe with pyloric atresia, or<br><br>and\or muscle dystrophy<br><br>EBS-PA	158684\131950	*PLEC*	AR	Rare form with generalized severe blistering widespread loss of skin with pyloric atresia, often fatal in infancy. In case of survival, skin fragility and nail dystrophy are life-long.	[219]

EBS intermediate with cardiomyopathy	508529\617294	Kelch like family member 24 - *KLHL24*	AD	Generalized blistering with cutaneous and follicular atrophy, linear and stellate scars, and hypopigmentation.<br><br>Cardiomyopathy with heart failure in young adulthood or later and may have lethal outcomes.	[220]
EBS localized with nephrotic syndrome (CD151 deficiency)	412189\300333	*CD151*	AD	Widespread blistering, particularly on pretibial areas, poikiloderma, nail dystrophy, loss of teeth, early onset alopecia, and esophageal webbing and strictures.	[221]

Supplementary TableS1. Epidermolysis Bullosa Simplex (EBS) group of disorders caused by mutations in different genes. The most prevalent forms are induced by mutation in keratin 5 and keratin 14 genes (*KRT5*, *KRT14*). Other forms are rare and include plectin *(PLEC*), exophilin 5 (*EXPH5*), Kelch-like member 24 *(KLHL24*), dystonin (*DST*), plakophilin (*PKP1*), beta-4-integrin (*ITG4*), CD151 antigen [2,44].
